# The impact of abdominal adiposity measured by sonography on the pulmonary function of pre-menopausal females

**DOI:** 10.1186/s40248-015-0018-z

**Published:** 2015-07-29

**Authors:** Zied Rasslan, Roberto Stirbulov, Roberto Saad Junior, Sergio Tercio Curia, Carlos Alberto da Conceição Lima, Eduardo Araújo Perez, Ezequiel Fernandes Oliveira, Claudio Ferdinando Donner, Luis Vicente Franco Oliveira

**Affiliations:** Santa Casa School of Medical Sciences in São Paulo, Rua Silvia, 301 apto 22 Bela Vista, CEP 01331-010 São Paulo, SP Brazil; Health Sciences of Santa Casa School of Medicine of Sao Paulo (FCMSCSP), Sao Paulo, Brazil; Nove de Julho University, Sao Paulo, Brazil; Mondo Medico, Multidisciplinary and Rehabilitation Outpatient Clinic, Borgomanero, NO Italy

**Keywords:** Abdominal adiposity deposition, Obesity, Respiratory function, Subcutaneous, Sonography, Visceral

## Abstract

**Background:**

The Body Mass Index (BMI) is a widely used parameter to study obesity; however it does not assess the distribution of body adiposity. Ultrasonography is a reliable method of measuring subcutaneous (SAT), visceral (VAT) and Total adipose tissue of the abdomen (TAT) to determine the influence of abdominal fat on pulmonary function by directly measuring abdominal adipose tissue.

**Methods:**

Eighty pre-menopausal, non-smoker, sedentary females with no history of pulmonary disease were subdivided into three groups: 25 normal-weight, 28 overweight, 27 obese. Absolute and predictive spirometric values were obtained: FVC, FEV_1_, FEV_1_/FVC, IC, ERV.

**Results:**

A positive correlation between increased %IC and decreased %ERV was observed with increased BMI (*p < 0.02; 0.001* respectively); %FVC, %FEV_1_ and %ERV decreased significantly as SAT (*p = 0.01, p = 0.02; p < 0.001*) and TAT (*p = 0.01, p = 0.03, p < 0.001*) increased, whereas VAT was negatively correlated only with %ERV (*p < 0.001*). Increments of 5 mm in TAT, VAT and SAT were followed by a reduction of 0.83 %, 0.81 %, 1.90 % in %FVC, respectively, as well as a reduction of 4.25 %, 4.31 % and 9.44 % in %ERV, respectively.

**Conclusions:**

Subcutaneous abdominal adipose tissue deposition in obese females has a greater negative influence on pulmonary function than visceral adipose tissue deposition.

## Background and Availability of supporting data section

Obesity has reached epidemic proportions in a number of Western countries and is considered as one of the main risk factors for the development of metabolic and cardiac diseases, permanent disability and death [1].

Increase in body weight is less relevant than distribution of the adipose tissue in the development of metabolic disorders [2]. Therefore, the frequently used Body Mass Index (BMI), which does not take into account such body fat distribution, is currently less accepted as a reliable method of evaluating the correlation between obesity and associated diseases [3]. Obesity, especially when concentrated in the abdomen and trunk, is associated with a number of co-morbidities. The main white adipose tissues are abdominal subcutaneous adipose tissue (SAT) and visceral adipose tissue (VAT) [4].

There are a number of imaging methods that assess body fat distribution, such as dual-energy x-ray, computed tomography, magnetic resonance, and sonography [5]. Sonographic assessment is a good alternative to other more sophisticated imaging techniques as it allows accurate, reliable, reproducible and cost-effective noninvasive collection of anthropometric data, with the added bonus of being painless and not exposing patients to ionizing radiation. This method also shows high correlation with tomographic findings [6].

The specific influence of obesity in respiratory disorders is complex and may depend on physical, mechanical, inflammatory and metabolic factors [7]. Obesity has been associated to dyspnea and asthma [8, 9]. The pulmonary function of obese females is influenced mainly by the amount and centripetal distribution of the excessive fat, which may interfere in the mechanics of the thoracic dynamics [10].

Pulmonary function abnormalities are more commonly seen in central obesity, however it is not completely clear if these abnormalities are solely a response to mechanical disturbances or if the changes in cellular metabolism and the byproducts produced by this central fat distribution also play a role in the respiratory problems [11].

In a very interesting study involving 14 obese men, Watson et al. hypothesized that thoracic expansion was reduced by the load imposed by increased total trunk fat volume or its distribution. However, the authors, using MRI analysis to assess internal and subcutaneous fat trunk and total abdominal and thoracic volumes at full inflation, failed to support the hypotheses that restriction of TLC, or impaired expansion of the thorax at full inflation in middleaged obese men, was simply the consequence of a large abdominal or total volume trunk fat volume or its distribution [12].

There are a number of respiratory changes observed in obese patients even in the absence of respiratory disease, such as changes in the respiratory mechanism, decreased muscle strength and gas changes, abnormal or borderline pulmonary function tests, and limited ability to perform exercises which are all directly related to increments in BMI [13–15].

The most common pulmonary function abnormalities reported in obese females are: a reduction in the residual expiratory volume (REV) proportional to the severity of obesity; and a reduction in vital capacity (VC) and total lung capacity (TLC) observed mostly in morbid obesity [16].

The magnitude of pulmonary function impairment is not always directly proportional to the severity of obesity, and may also be influenced by gender and distribution of the adipose tissue [17, 18]. The majority of obese females present spirometry values within normal predictive ranges. Even though several studies have addressed the influence of obesity on pulmonary function, only a small number have focused on the effect of body adipose tissue distribution [15, 19, 20].

The influence of body adipose tissue distribution on pulmonary function of individuals who are overweight or mildly obese also remains unclear; only a small number of studies with this population included measurements of central adiposity, finding a decreased pulmonary function in non-obese individuals [18, 20–22].

The objective of the current study is to assess the effects of abdominal subcutaneous and visceral adipose tissue deposition on the spirometric parameters of obese females comparing them to normal-weight and overweight females.

## Methods

Following approval of the Ethics Committee on Human Research of Santa Casa de São Paulo a total of 80 volunteer adult sedentary females who fitted the inclusion and exclusion criteria were consecutively enrolled at a tertiary teaching institution. All the participants consented to participate in the study after properly informed.

In order to be considered sedentary, participants should report physical activity equivalent to less than a 30-min walk three times a week.

To be included in the study females should also be pre-menopausal, over 22 and under 47 years of age, and have no past history of cardiac and respiratory diseases, as well as be non-smokers and have no recent respiratory complaints. Females who reported the use of bronchodilators or broncho constricting drugs, as well as those with systemic diseases or who underwent surgery in the 6 months prior to entering the study protocol were excluded.

All the participants were interviewed and responded to a guided questionnaire that contained general epidemiological data and specific respiratory data. Weight (in kilograms) and height (in meters) were obtained to determine the BMI, which was used to establish obesity.

The participants were subdivided into three distinct groups according to their body mass index: 25 females with normal body weight (BMI ranging from 19 to 24.9 Kg/m^2^); 28 overweight females (BMI ranging from 25 to 29.9 Kg/m^2^); and 27 obese females (BMI greater than 30 Kg/m^2^).

### Abdominal adiposity distribution

Abdominal adiposity distribution was then assessed using sonography. Three measurements were obtained: subcutaneous adipose tissue (SAT), visceral adipose tissue (VAT), and total adipose tissue of the abdomen (TAT = SAT + VAT). All sonography examinations were performed by the same experienced physician (co-author) using an Image point Hewlett Packard, Chicago, USA and a 7.5 megahertz transducer. Volunteers were instructed to fast for 12 h prior to the examination, which was then performed in the supine position under natural breathing. The transducer was placed longitudinally under pressure in the midline of the abdomen at the level of the supra-umbilical mesogastric area. The total thickness of the abdominal adipose tissue, as well as that of the subcutaneous and visceral adipose tissue was determined from frozen images and expressed in centimeters (cm). Subcutaneous adipose tissue thickness was calculated by measuring the distance between the skin and the anterior portion of the rectus abdominalis muscle. Visceral adipose tissue thickness was established by measuring the distance between the posterior wall of the rectus abdominalis muscle and the posterior wall of the aorta at the level of the abdominal aorta bifurcation at the xyfo-umbilical line. Total abdominal adipose tissue was determined by adding the latter two measurements.

### Pulmonary function

Pulmonary function was assessed with a Koko spirometer associated to a pneumotacograph (nSpire Health Inc, USA). All spirometric examinations were performed by the same experienced pneumologist (co-author) in a quiet and calm environment, with controlled temperature and humidity, from 8:00 am to 12:00 pm, in order to avoid circadian influences.

Before commencing the spirometric test participants rested 5 to 10 min and then were carefully instructed on the techniques and objectives of the test. Spirometric data were collected in the sitting position using a nasal prong and standard protocol [23].

Both the absolute and the percent predictive values of the following spirometric parameters were studied: Forced Vital capacity (FVC), Forced Expiratory Volume in 1 ° second (FEV_1_), the proportion between FEV_1_ and FVC (FEV_1_/FVC ratio), Inspiratory Capacity (IC), and Expiratory Reserve Volume (ERV). In this study, we have considered the difference between Vital Capacity and Inspiratory Capacity as the extrapolated value of ERV.

Both forced and non-forced spirometries were performed for the determination of the time-volume and flow-volume curves. The non-forced spirometry was performed to obtain the pulmonary capacities and volumes. The volume-time curve obtained from forced spirometry was determined by selecting the best of three acceptable runs and was used to extract the percent predictive Forced Vital Capacity (%FVC), and the Forced Expiratory Volume in 1 ° second (%FEV_1_) [24]. The flow-volume curve was also submitted to criteria of acceptability and adopted predictive values recommended by the ATS [23].

The percent predictive IC and ERV were correlated to pairs of groups (normal-weight vs overweight; normal-weight vs obese; overweight vs obese).

Results were tabled and analyzed statistically using a commercially available software package (Statistical Package for Social Science version 17.0). Parametric data were analyzed using Spearman’s correlation test in order to try to establish any possible correlation between BMI, total abdominal adipose tissue, subcutaneous adipose tissue, visceral adipose tissue, and spirometric parameters. Kruskal-Wallis test was used to establish differences among the three BMI groups for the studied parameters. When a statistically significant difference was found, the variables were treated with Mann–Whitney to compare paired groups. Multivariance regression tests were used to study the influence of qualitative and quantitative variables on the reduction of the ERV. Variables were expressed in mean values and standard deviations with a significance level established at *p* < 0.05.

## Results

All the studied obesity parameters (weight, BMI, TAT, SAT, VAT) were significantly different among the three BMI groups (normal-weight, overweight and obese) (*p* < 0.001) (Table 1).

**Table 1:** Anthropometric, sonographic and spirometric data of the studied population of 80 sedentary volunteer females

Variable	Normal-Weight	Overweight	Obese	
	Mean ± SD	Mean ± SD	Mean ± SD	*p*
n	25	28	27	
Age (years)	31.2 ± 7.11	31.9 ± 6.85	32.9 ± 7.25	NS
Height (cm)	1.62 ± 0.09	1.63 ± 0.06	1.59 ± 0.08	NS
Weight (Kg)	59.1 ± 7.01	73.7 ± 6.12	84.5 ± 10.6	<0.001
BMI (kg/m^2^)	22.4 ± 1.57	27.8 ± 1.25	33.3 ± 2.18	<0.001
SAT (cm)	1.71 ± 0.38	2.52 ± 0.61	3.13 ± 0.65	<0.001
VAT (cm)	3.17 ± 0.53	4.04 ± 1.03	5.04 ± 1.47	<0.001
TAT (cm)	4.89 ± 0.68	6.56 ± 1.18	8.17 ± 1.54	<0.001
%FVC	102.5 ± 11.6	104.2 ± 12.2	100.6 ± 10	NS
%FEV_1_	99.4 ± 9.46	101.5 ± 12.1	99 ± 8.71	NS
FEV_1_/FVC	0.84 ± 0.04	0.83 ± 0.04	0.85 ± 0.05	NS
%IC	113 ± 16.2	125.9 ± 18	123 ± 12.8	0.02
%ERV	94.4 ± 30	79.6 ± 25.8	64 ± 25.7	0.001

*%FVC*, percent predictive Forced Vital Capacity, *%FEV*_*1*_, percent predictive Forced Expiratory Volume in first second, *SD*, Standard Deviation, *%IC*, percent predictive Inspiratory Capacity, *%ERV*, percent predictive Expiratory Reserve Volume, *BMI*, Body Mass Index, *NS*, non-significant, n, number of individuals, statistical significance (*p* < 0.05), *BMI*, Body Mass Index, *TAT*, Total abdominal adipose tissue, *VAT*, Visceral adipose tissue, *SAT*, Subcutaneous adipose tissue

**Fig. 1:**
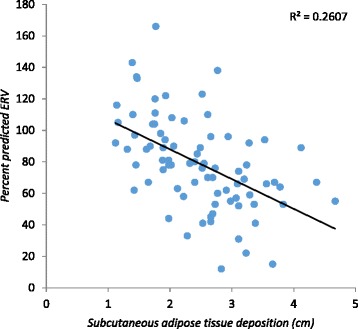
Correlation between subcutaneous adipose tissue and the percentage of predictive values of the Expiratory Reserve Volume (%ERV)

**Fig. 2:**
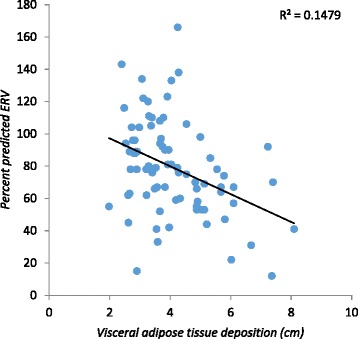
Correlation between visceral adipose tissue and the percentage of predictive values of the Expiratory Reserve Volume (%ERV)

**Fig. 3:**
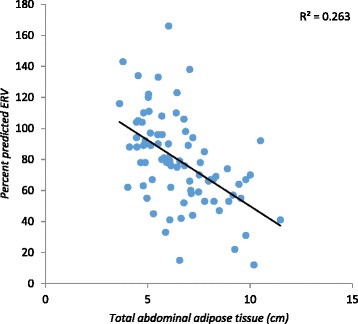
Correlation between total abdominal adipose tissue and the percentage of predictive values of the Expiratory Reserve Volume (%ERV)

The percent predictive ERV was significantly reduced in the three groups (*p* = 0.001), whereas the percent predictive IC was significantly increased in these groups (*p* = 0.02). The percent predictive FVC and FEV_1_ presented a significant negative correlation with SAT (r – 0.26; *p* = 0.019 and r – 0.24; *p* = 0.026), respectively (Table 2). No significant correlation was found between FEV_1_/FVC and %IC and SAT (r = 0.15; *p =* 0.18 and r = 0.21; *p =* 0.058, respectively) or VAT (r = 0.01; *p =* 0.86 and r = 0.09; *p =* 0.42, respectively) (Table 2). The percent predictive ERV presented a significant negative correlation with subcutaneous, visceral and total abdominal adipose tissue (r – 0.56; *p* < 0.001 and r – 0.36; *p =* 0.001 and r–0.54; *p* < 0.001 respectively) (Figs. 1, 2 and 3). The percent predictive FVC and FEV_1_ also showed a significant negative correlation with total abdominal adipose tissue (r- 0.27; *p* = 0.015 and r- 0.24; *p* = 0.031, respectively) and subcutaneous adipose tissue (r- 0.26; *p* = 0.019 and r- 0.24; *p* = 0.026, respectively) (Table 2). In our study, we have realized that age and height are possible bias, so correlated with the percentage of predicted. The correlation with the absolute values and the results were not positive, as expected.

**Table 2:** Correlation between spirometric parameters and abdominal adipose tissue (total, subcutaneous and visceral) measurements of the 80 studied pre-menopausal females

Variable	Subcutaneous Adipose Tissue		Visceral Adipose Tissue		Total Abdominal Adipose Tissue	
	r	*p*	r	*p*	r	*p*
%FVC	−0.26	0.01	−0.20	0.06	−0.27	0.01
%FEV_1_	−0.24	0.02	−0.17	NS	−0.24	0.03
FEV_1_/FVC	0.15	NS	0.01	NS	0.09	NS
%IC	0.21	0.058	0.09	NS	0.18	NS
%ERV	−0.56	<0.001	−0.36	0.001	−0.54	<0.001

*%FVC*, percent predictive Forced Vital Capacity, *%FEV*_*1*_, percent predictive Forced Expiratory Volume in first second, *%IC*, percent predictive Inspiratory Capacity, *%ERV*, percent predictive Expiratory Reserve Volume, *NS*, non-significant, *r*, Pearson’s correlation coefficient

When observing the paired groups (normal-weight vs overweight; normal-weight vs obese; overweight vs obese), the variable percent predictive IC presented a significant positive correlation with the pairs normal-weight vs overweight and normal-weight vs obese. For the variable percent predictive ERV values a significant negative correlation was observed in all three pairs (Table 3).

**Table 3:** Comparison between pairs of BMI groups according to percentages of predictive values of the Inspiratory Capacity and Expiratory Reserve Volume

Variable	Pairs of Groups		
	Normal-weight	Normal-weight	Overweight
	vs.	vs.	vs.
	Overweight	Obese	Obese
%IC	0.01^a^	0.02^a^	NS
%ERV	0.04^a^	0.001^a^	0.04^a^

^a^Statistical significance on Mann–Whitney test; *NS*, non-significant, *%IC*, percentage of predictive Inspiratory Capacity, *%ERV*, percentage of predictive Expiratory Reserve Volume

Increments in 5 mm in total abdominal adipose tissue, visceral adipose tissue, and subcutaneous adipose tissue reflected on a drop of 0.83 %, 0.81 %, and 1.90 % in the percent predictive FVC, respectively (Table 4). The same was observed for percent predictive ERV values, which dropped 4.25 %, 4.31 %, and 9.44 %, respectively following 5mm increments in total abdominal adipose tissue, visceral adipose tissue and subcutaneous adipose tissue (Table 4).

**Table 4:** Effects of 5mm increments in abdominal adipose tissue on the percentage of predictive values of the Forced Vital Capacity and the Expiratory Reserve Volume in the studied population

5mm increment in adipose tissue deposition	Reduction	
	%FVC	%ERV
TAT	0.83 %	4.25 %
VAT	0.81 %	4.31 %
SAT	1.90 %	9.44 %

*%FVC*, percentage of predictive Forced Vital Capacity, *%ERV*, percentage of predictive Expiratory Reserve Volume, *TAT (cm)*, Total abdominal adipose tissue, *VAT (cm)*, Visceral adipose tissue, *SAT (cm)*, Subcutaneousadipose tissue

A multivariate analysis was used to compare BMI with significant reductions in the percentages of the predictive values of ERV in overweight and obese females.

Increments in subcutaneous and total adiposity were followed by a significant reduction in the percentages of predictive values of ERV in overweight (*p* = 0.001 e 0.009) and obese females (*p* < 0.001). Increments in visceral adiposity thickness were followed by a significant reduction in the ERV of obese females (*p* = 0.007).

The multivariate analysis showed that in obese females subcutaneous, visceral and total abdominal fat depositions increase the risk of reduction in the percentages of predictive values of ERV in over 14, 9, and 25 times, respectively (Table 5). In overweight females, subcutaneous and total abdominal fat depositions increase the risk of a reduction in the percentages of predictive values of ERV in over 10 and 4 times, respectively (Table 5).

**Table 5:** Results of multiple regression analysis for the percent predictive ERV including body mass index and sonographic measurement (subcutaneous, visceral and total abdominal adipose tissue)

Variable	OR	IC 95 %	*p*
BMI			
Normal weight ≤ 24.9			
Overweight	3.96	1.25-12.6	0.010
Obese	9.00	2.55-31.2	0.001
SAT			
Normal weight ≤ 2.09			
Overweight	10.7	3.36-34.4	<0.001
Obese	14.9	3.27-66.6	<0.001
VAT			
Normal weight ≤ 3.7			
Overweight	1.33	0.48-3.67	0.570
Obese	9.25	1.85-45.4	0.007
TAT			
Normal weight ≤ 5.57			
Overweight	4.38	1.45-13.3	0.009
Obese	25.6	4.71-142.8	<0.001

*OR*, odds ratio, *IC*, confidence interval, *p*, significance, *BMI*, Body mass índex, *TAT*, Total abdominal adipose tissue, *VAT*, Visceral adipose tissue, *SAT*, Subcutaneous adipose tissue

## Discussion

The current study was motivated by the knowledge that adipose tissue distribution is essential for determining the effects of obesity on the mechanics of the respiratory system. BMI alone is not reliable to determine specific risk factors for the development of associated diseases in obese patients, as it does not account for the distribution of body adiposity [21]. In the current study the comparison of abdominal adipose deposition (total abdominal adipose tissue, visceral adipose tissue and subcutaneous adipose tissue) with the pulmonary function of normal-weight, overweight, and obese premenopausal females revealed significant differences in all the spirometric values studied.

Although a number of studies have also observed an inverse correlation between respiratory function and obesity, as well as specific body adiposity distribution [10, 22, 25], the current study is, to authors’ knowledge, the first one to correlate abdominal wall thickness measured by sonography with its subdivisions (visceral and subcutaneous) to spirometric values. In accordance with the literature [10], no significative spirometric differences were observed among normal-weight, overweight and obese females. However, when abdominal adiposity distribution was correlated to spirometric values in this otherwise healthy sample of females, a significant negative correlation was found, suggesting that abdominal adipose tissue deposition (subcutaneous and total) interferes in the capacity of sustaining the maximum expired volume after maximum inspiration. These findings suggest that total abdominal adipose tissue and subcutaneous adipose tissue may predict a reduction in spirometric values in this population.

The subcutaneous adipose tissue deposition in females most likely determines a limitation in the mobility of the abdominal and thoraco-abdominal muscles. Visceral adipose tissue deposition, on the other hand, is expected to limit diaphragmatic mobility. Association of both SAT and VAT deposition may also lead to restricted lung expansion during inspiration and reduction in the caliber of the peripheral airways causing abnormalities in perfusion/ventilation and developing hypoxemia [26]. These abnormalities may explain the higher prevalence of respiratory problems in obese females, particularly when under exertion or in the supine position (sleeping or under anaesthesia) [26].

It is also important to determine if there is a specific pattern of fat deposition that is most likely to cause the changes described above. The females who participated in the current study were all pre-menopausal. In this stage adipose tissue distribution tends to be more peripheral (femoral and gluteous) than abdominal. Interestingly, the studied population showed that total fat, both visceral and subcutaneous depot influenced ERV in a statistically significant manner (Table 2). By studying increments of 5mm in adipose tissue distribution, it was observed that subcutaneous adipose tissue increments were the ones with the most significant negative correlation with pulmonary function parameters such as FVC and ERV (Table 4).

A number of factors are involved in the pulmonary abnormalities found in obese females, other than the mechanical ones described above. Abdominal adiposity has been correlated to insulin resistance, abnormalities in the metabolism of glucose, hypertension and dyslipidemia, all known conditions associated with the metabolic syndrome [27]. Females present significantly higher leptine levels than males with the same BMI, suggesting that this peptide may play a role in gender differences found between adipose tissue deposition and respiratory function [28]. Dysregulation of adipokine secretion, free fatty acid toxicity, and the site-specific differences in abdominal (visceral) versus subcutaneous adipose tissue support abdominal obesity as a causal factor mediating the insulin resistance, increased risk of diabetes, and cardiovascular disease in the metabolic syndrome [29].

The study by Torchio et al. suggests that obesity and airway responsiveness are associated. This is true in both sexes. In men, the relationship appears to be linked to the effects of obesity on lung volumes and breathing control. In women, the relationship is presumably more complex and possibly involves additional biological/biochemical mechanisms other than breathing control [30].

When subdividing the studied population into groups according to weight and comparing them two by two (normal-weight vs overweight; normal-weight vs obese; overweight vs obese) the reduction in the %ERV and the increase in the %IC, following increments in BMI, sustained the findings of the overall group.

No significant differences were found in the %FVC and %FEV_1_ among the original three BMI groups (normal-weight, overweight, and obese). According to Harik-khan et al. [17], it is the subcutaneous fat deposition in the upper thorax rather than abdomen that determines the greatest impact on respiratory function. However, in the current study subcutaneous adiposity and total abdominal adiposity were found to have a significant negative impact on respiratory function of the obese females.

It is very important speculate that the main abnormalities in obesity are the transmission of the high pressures in the intra-abdominal compartment to the thorax that dramatically reduce functional residual capacity and expiratory reserve volume and oblige patients to breathe on the flatter, less efficient, part of their pressure-volume curve, increasing the work of breathing.

Steier et al. [31] conducted an observational study of lung volume and elasticity in nine obese and nine regular weight subjects, seated and supine, during spontaneous breathing. Seated and supine total lung capacity (TLC) and subdivisions were measured by multibreath helium dilution method. Using balloon catheters, oesophageal and gastric pressures were recorded. Transpulmonary pressure was calculated by the mouth pressure minus oesophageal pressure, and complete expiratory volume curves transpulmonary pressure were measured. The obese subjects were more restricted than the normals, had dramatically reduced expiratory reserve volume and end-tidal functional residual capacity (FRC) was smaller when seated, but was similar when supine. Gastric pressures at FRC were significantly elevated in the obese, as were the end-expiratory oesophageal pressures at FRC. BMI correlated with end-expiratory gastric and oesophageal pressures. Therefore, according to authors obese subjects have markedly increased gastric and oesophageal pressures, both when upright and supine, causing dramatically reduced FRC and ERV, which increases work of breathing.

In 2009 Leone et al. also observed a positive correlation between pulmonary dysfunction and metabolic syndrome in females, independent from other cardiovascular risk factors or even BMI, especially in those with abdominal adiposity [20]. Metabolic syndrome and abdominal adiposity are associated with a restrictive respiratory pattern, where the greater the abdominal circumference the worse is the FVC [20, 30]. Although literature considers visceral adipose tissue deposition to cause a greater metabolic, mechanical and hormonal effect on pulmonary function than subcutaneous adipose tissue deposition [30, 32], this did not seem to be the case in the population of overweight and obese females studied in the current series. Visceral adipose tissue deposition most likely plays an important role in males, but seems to have less impact on females; in the latter, sonography shows a greater sensitivity for determining abdominal adoposity [29]. It also seems the speed of weight gain may play a role in the development of respiratory impairment, as females that gain weight slowly over time do not present significant spirometric changes, suggesting that the body develops adaptive mechanisms to the excessive adipose tissue.

In 2008 Sutherland et al. studied the effect of adiposity measured by dual-energy x-ray absorptiometry on lung function in healthy adults finding that BMI was only weakly associated with pulmonary volumes [32]. However, all the indicators of localized body fat deposition presented a significant negative correlation with FVC and ERV in both males and females, but especially in females. Some studies suggest that obesity is a risk factor for small airways disease and may be associated to exercise intolerance found in overweight and obese healthy adults [33].

According to Farah et al. [34], the obese asthma phenotype is increasingly encountered in clinical practice. Epidemiological data indicate that obesity increases the prevalence and incidence of asthma, and evidence that obesity precedes the development of asthma raising the possibility of a causal association. Despite more than a decade of research into this association, the exact mechanisms that underlie the interaction of obesity with asthma remain unclear. It is unlikely that the asthma-obesity association is simply due to comorbidities such as obstructive sleep apnea or gastroesophageal reflux disease.

The clinical implication of weight gain is an overload in the respiratory muscles caused by a superior displacement of the diaphragm, which determines a decrease in the ERV and FRC, and may lead to dyspnea upon exertion in obese women [18].

Aguiar et al. [35] in a study involving 16 obese men and women undergoing bariatric surgery, observed a significant improvements of FVC (*p* = 0.002) and FEV_1_ (*p* = 0.003) after bariatric surgery. Analyzing the ventilatory maximum pressures only in obese women in the preoperative period, the authors observed a significant reduction of maximum inspiratory pressures (52.67 ± 18.91) and maximum expiratory pressures (53.58 ± 16.88) in comparison to the reference values and a significant increase in pressures occurred in the postoperative period (MIP = 0.002 and MEP = 0.001). Therefore, the authors concluded that the reduction of adipose tissue caused by bariatric surgery effectively increases maximum ventilatory pressures and pulmonary function in patients with severe obesity.

The current findings of decreased pulmonary function associated to increased subcutaneous and visceral adipose tissue deposition in overweight and obese females corroborate the above studies.

## Conclusions

The authors conclude that the progressive gain in BMI and the pattern of abdominal adipose tissue distribution, especially subcutaneous adipose tissue, interfere with the ERV of overweight and obese females. Further studies with larger populations and a longitudinal design are needed to better clarify these findings.
